# “The questions made me realize how many times I could have been saved and removed from that situation”: The experiences of patients attended to by paramedics for intimate partner violence, and actionable implementations for paramedicine

**DOI:** 10.1186/s12905-025-03753-9

**Published:** 2025-05-26

**Authors:** Rory A. Marshall, Nicole Merritt, Tori N. Stranges, Stephen Bartlett, Simon Sawyer, Paul van Donkelaar

**Affiliations:** 1 School of Health and Exercise Sciences, Faculty of Social Development, University of British Columbia Okanagan, 1147 Research Road, Kelowna, BC V1V 1V7 Canada; 2 British Columbia Emergency Health Services, 101-1515 Keehn Road, Kelowna, BC V1X 5T3 Canada; 3 School of Clinical Sciences, Faculty of Health, Queensland University of Technology, 2 George Street, Brisbane, QLD 4000 Australia; 4 School of Paramedicine, Faculty of Medicine, Dentistry and Health, Griffith University, 1 Parklands Drive, Southport, QLD 4215 Australia

**Keywords:** Intimate partner violence, Paramedics, Paramedicine, Healthcare pathways, Public health, Healthcare access

## Abstract

**Introduction:**

Intimate partner violence (IPV) persists as a severe and prevalent criminal, social, and health issue, most commonly affecting women. Survivors of IPV frequently engage with the healthcare system to access treatment, support, and resources. Paramedics commonly encounter, either knowingly or unknowingly, patients experiencing IPV. There is little empirical research on how paramedics manage cases involving IPV. Objective: To examine how the perspectives and experiences of survivors of IPV who have been attended to by paramedics can inform our understanding of paramedic services.

**Methods:**

Using an interpretive description qualitative approach in the context of paramedicine, self-identified women (18+ years) who reported a history of IPV and being attended to by paramedics for IPV-caused reasons participated in semi-structured interviews. Interviews were transcribed verbatim and de-identified. De-identified transcripts were inductively analyzed (NVivo) for common patterns.

**Results:**

*N* = 9 survivors participated in interviews. Participants experienced cyclic and escalating physical, sexual, psychological, and coercive control forms of IPV. Participants primarily reported accessing paramedic services following instances of severe IPV and reported receiving minimal treatment and support. Challenges included bias and discrimination, poor individual paramedic conduct, undereducated and undertrained paramedics, insufficient infrastructure, inadequate transitions into healthcare and community services, perpetrator dynamics, and survivor dynamics. Corresponding solutions were safe and equitable paramedic behaviour, respectful conduct, mandatory education and training, develop functional infrastructure, develop functional transitions, and utilize techniques to engage with perpetrators and survivors.

**Conclusion:**

Personal, situational, practitioner, paramedic service, and broad systemic infrastructure challenges cause survivors of IPV to be underserviced by paramedic services. Inadequate intervention efforts may be harmful or fatal for survivors. Survivor-derived solutions may guide paramedic service improvements. With improved service delivery, paramedics could evolve into reliable and useful resources for survivors of IPV.

**Supplementary Information:**

The online version contains supplementary material available at 10.1186/s12905-025-03753-9.

## Background

Intimate partner violence (IPV; i.e., *dating violence*,* partner abuse*) is a preventable pandemic. IPV encompasses the use of physical, sexual or psychological behavioural aggressions, and/or stalking behaviours by a current or former intimate partner to cause harm, and exert power and control [1]. Given the global life-time prevalence of IPV among women of one in three and a 12-month prevalence of one in ten [2, 3], as well as the cyclic and escalating nature of IPV [1, 4], the implications for the general population when applying the incidence rates remain dire. The detriment to the health and well-being persists as a harm for survivors and family members in the community. While IPV affects people from all walks of life, women, girls, gender diverse people, and vulnerable populations bare the greatest risk of experiencing IPV [5, 6]. 

IPV is well documented to cause harmful acute and chronic health consequences [7–9]. As such, IPV may cause survivors to attempt to access the healthcare system for care, treatment, support, and/or referral. IPV sequela may create barriers for survivors attempting to access the healthcare system [10]. The emergency department is commonly thought of as the first point of contact for survivors utilizing the healthcare system [11]. A proactive and preventative approach to IPV is proposed as a superior method compared to current reactive responses [12, 13]. For example, educating youth and creating a positive culture around healthy relationships is an upstream approach targeted to prevent IPV among youths, ideally translating into adulthood [13, 14]. However, complex systems of privilege and oppression intersecting with social determinants of health often amplify challenges in prevention efforts yielding a reliance on reactive processes [12, 15]. Modern healthcare, especially healthcare accessed through hospital emergency departments, is commonly built on reactive emergency medicine ideologies and pathways to care [16]. Of course, many services operate outside of the emergency department. Social workers commonly encounter survivors and perpetrators of IPV, however, outside of specialist teams, may lack the appropriate healthcare and systems knowledge to facilitate referrals to healthcare and resources [17]. Social workers often have strong, interconnected networks of non-healthcare resources for survivors of IPV that can assist in the overall support of survivors beyond physiological healthcare alone [18]. Police services also commonly encounter survivors and perpetrators of IPV. Even when survivors are recognized and transported to the emergency department by police, survivors remain undertreated for the constellation of issues caused by IPV, and presumably underserviced, by the healthcare system [19]. Further, IPV-caused brain injury is common among survivors and may further exacerbate challenges for survivors [10, 20–23]. Patients presenting to the emergency department are unlikely to be screened for, documented as, or recognized as experiencing IPV [24–28]. Thus, an opportunity may exist for an interdisciplinary service provider to be mobilized in a novel sense as an expert resource to meet address the needs of and advocate for survivors of IPV.

Paramedics (i.e., *emergency medical technicians*, etc.), mobile healthcare and emergency service professionals, are uniquely positioned to serve survivors of IPV through integrated community and healthcare system pathways [29]. The inherently multifaceted, versatile, and interdisciplinary nature of the paramedic role plays to the strengths of this health profession, including the privilege to be invited into patients’ homes and lives, gaining insight and context which can be otherwise lost to the healthcare system. A typical paramedic service response to a 911 call is initiated by a call to an emergency medical call taker. The emergency medical call taker attains enough information to advance the service request to a dispatcher. The dispatcher then assigns the appropriate resources based on the information from the call taker. Paramedics then attend the location and conduct patient care on scene. Next, paramedics discuss options with the patient, namely conveyance to a healthcare facility (e.g., emergency department, urgent care) or non-conveyance (e.g., the patient remains in community). If a patient chose to remain in community, they may or may not be referred to another resource. If a patient is conveyed to a healthcare facility, then paramedics continue to provide patient care and position the patient to move through the healthcare system as is most appropriate based on the working diagnosis. Paramedics have been shown to effectively identify non-traditional healthcare needs and initiate referrals, leading to appropriate healthcare utilization [30]. Further, as interdisciplinary providers, paramedics are well situated to understand the broader aspects involved in the health and well-being of survivors of IPV. The criminal and social components of situations involving IPV may not be lost on paramedics, allowing them to recognize the larger picture through a healthcare lens.

While great opportunity exists for paramedics, the current state of paramedicine presents challenges in embracing this role. In Canada, the Paramedic Association of Canada is the primary governing body for paramedic educational and vocational competencies [31]. However, no iteration of the National Occupational Competency Profile includes IPV [31, 32]. Despite the inclusion of competencies for child abuse, elder abuse, abuse of persons with disabilities, and abuse of bariatric persons, IPV or partner abuse remains omitted [31]. Minute pockets of guidance for individual services or areas for situations involving IPV may exist, but on a national scale there are no educational, training, or vocational standards for paramedics encountering situations involving IPV. Thus, it is currently likely paramedics may not be in a position to serve as expert resources for survivors.

Many survivors access the healthcare system who have acutely been experiencing IPV both pre- and post- substantial injury, highlighting multiple contact points and an opportunity to intervene [19]. Importantly, the risk of homicide among survivors is increased when attempting to exit a violent relationship [33]. Ergo, it is paramount that healthcare system engagement is appropriate and supports survivors towards their desired outcomes, co-creating safe and reasonable pathways to escape violence. Without intervention and ongoing prevention to break repeated cycles of violence, continued violence and increased risk of the progression to homicide are likely to persist.

The aptitudes of healthcare providers and students have been examined by different tools, typically highlighting deficiencies that cannot be addressed by education or training alone [34–37]. For example, among surveyed paramedics practicing in Canada, the desire to help survivors of IPV and knowledge of IPV were well scored constructs [38]. However, self-efficacy and emotional readiness were poorly scored constructs [38]. This indicates that complexities beyond traditional trains of thought exist in these multifaceted situations. In this sample, it appeared that paramedics wanted to help survivors and were knowledgeable about IPV but lacked the applicability of what to do when encountering survivors and the emotional capacity to meaningfully engage. A common solution of education or training may be useful component but should be tailored to the identified needs.

The survivor perspective when in the role of the patient is one that has yet to be explored when examining the intersection of IPV and paramedicine. It would be ill-informed and ignorant to continue to pursue knowledge in this field without learning from those with lived experience. As such, the purpose of this study was to explore the intersection of IPV and paramedicine from the service user perspectives of survivors of IPV. The objective of this study was to examine how the perspectives and experiences of survivors of IPV who have been attended to by paramedics may inform the understanding of current clinical practice and guide potential improvements for paramedicine.

## Methods

### Ethical approval and research quality

This study was reviewed, approved, and conducted in accordance with the permission of the University of British Columbia Okanagan Behavioural Research Ethics Board (#H22-02770). All participants provided written informed consent prior to enrolment in the study. This research was conducted ethically in accordance with the Declaration of Helsinki.

### Approach, positionality, and safety

This research was conducted under a pragmatic philosophical worldview, relativist ontology, and constructivist epistemology. With this in mind, this study was purposefully conceptualized, designed, and conducted using an inductive interpretive description (ID) qualitative approach specific to the research objective and context within the applied discipline of paramedicine [39]. ID is not a step-by-step cook book approach to conducting research but rather an adaptable constructivist-rooted epistemological approach which was consciously applied in alignment with the research objectives and worldview [39]. Commonly, the experience of the researchers may be viewed as bias that requires methodological elimination from study design, but in ID this experience is a valuable instrument of the co-created research between the researchers and the participants [40]. The researchers’ technical knowledge, research experience, and personal background are commonly edifying to the project [40]. 

The research team was composed of clinician-researchers (RAM, NM, SB, SS) and researchers (TNS, PvD). These roles combined with the unique and diverse positionality of the research team were cumulatively leveraged to enhance the undertaking in alignment with the objectives of this work.

In addition to the contributions of the research team, this project was designed and conducted with active engagement and partnership with community partners who provide services to survivors of IPV. While the use of knowledge translation in paramedic research remains limited, using established knowledge translation and co-created research approaches in paramedic-led research would likely improve practical applicability and utility [41]. Co-created research involves adhering to the principles of co-created research in the context of each project by using practical strategies to engage with those principles [42]. The integrated knowledge translation guiding principles were applied to the partnered aspects of this research [43]. The community partners engaged in the research production process at mutually agreed upon instances throughout the research process. Trust, respect, dignity, transparency, communication, shared decision making, and mutually beneficial participation were core principles used in this research.

Participant safety was the first priority in all decision making. All procedures, and operational plans were reviewed and critically revised for safety and sensitivity considerations by our community partner organizations. As the frontline experts who work with the participant population of this project on a daily basis during acute crisis through ongoing supportive work and advocacy, the needs of survivors were constantly prioritized through partnership consultations. All research was positioned to be conducted in the most appropriate, informative, and sensitive manner that also accommodated achieving the research objectives.

### Participants

To address the primary objective of this study (to examine how the perspectives and experiences of survivors of IPV who have been attended to by paramedics may inform the understanding of current clinical practice and aspirations for future clinical practice), culturally and linguistically diverse women (18 + years) who felt comfortable participating in the research in the medium of English, and who had been attended to by paramedics in the Canadian provinces of Alberta, British Columbia, or Saskatchewan for IPV-caused reasons were eligible to participate in this study. There was no recency requirement for participants on when the interaction with paramedic services occurred. This region was selected because each province has similar population distribution (i.e., *large centers and small centers spaced over a large geographical area*), geographical proximity, demographic makeup, paramedic education and training, and paramedic scope.

### Interview recruitment

Community partners (e.g., women’s shelters, counselling services) and associated connections (e.g., formal and informal networks) distributed a combination of promotional efforts (e.g., physical posters, emails, snowball sampling; efforts tailored to the request of each partner) to direct potential participants to an online survey. The survey was composed of a consent form, inclusion criteria items, safe contact information items, demographic items, and a free text box. Respondents who met the inclusion criteria were re-directed to a separate survey than those who did not meet the inclusion criteria. Non-eligible respondents remained on the initial survey without contact information items. Seamless integration of the two surveys allowed for potentially sensitive information (e.g., history of IPV) to be stored separately from identifying information (e.g., contact information) for eligible respondents. Although the inclusion criteria may have limited otherwise eligible respondents (e.g., non-English speaking individuals, etc.), the opportunity to gather their valuable perspectives was accommodated in the free text box within the non-eligible survey. Analysis of the free text responses from participants who were not eligible for interviews was not included in this study. This decision was made to avoid conflating interview data about the perspectives and experiences of a known group (i.e., *described by the eligibility criteria*) with perspectives and experiences outside that group.

A member of the research team established a safe method of communication with respondents, confirmed eligibility, confirmed retained interest in study participation, and provided the interview consent form. Participants were encouraged to review the consent form and report to the research team with any questions, comments, or concerns. The consent form outlined that interviews were to be video and audio recorded. If participants wished to participate after reviewing the consent form and having any questions, comments, or concerns addressed, then participants were scheduled for online video interviews (University of British Columbia-licensed Zoom, San Jose, USA).

### Interviews

Individual interviews were selected as the best method for the objectives of the project. Video interviews were selected to accommodate the privacy, safety, circumstances, and locations of participants. Under specific guidance from our community partners, video interviews were chosen over audio interviews to attempt to mitigate safety concerns and to denote any specific body language. Although in person interviews may have allowed for a more natural conversational dynamic, the inherent benefits of virtual video interviews outweighed the limitations.

The interviews were semi-structured under the guidance of a flexible interview guide. The interview guide focused on five main areas: 1) experience with violence, 2) experience with paramedics in the context of violence, 3) desired experience and services from paramedics, 4) challenges when accessing and utilizing paramedic services, and 5) solutions for identified challenges. Prior to each interview, standard operating procedures were used to mitigate safety concerns and ensure informed consent, reminding participants that interviews were video and audio recorded. Participants were encouraged to retain a copy of the consent form. Interviews were approximately 45 minutes in length. All interviews were conducted by a single researcher who also kept field notes (NM). Interviews were video (where possible; participants retained the autonomy to decline video recording) and audio recorded (Zoom). All interviews were, to our knowledge, conducted without incident. No crisis, safety concerns, or psychological upset were noted or reported during, immediately following, or during post-interview follow up. Participants were compensated with a $50.00 honorarium for their participation.

A hybrid of automated transcription (Zoom), line-by-line manual transcript confirmation, and descriptive field note integration (e.g., body language, tone, etc.) were purposefully used to meaningfully engage with the data beyond the use of automated transcription alone [39], and remove any identifying information from each transcript.

### Analysis

Demographic items for individuals who participated in interviews (collected from the recruitment and eligibility survey) were pooled and presented as basic proportions of the overall sample (mean ± standard deviation or % (n/N); as appropriate). Pooling the data and not having demographics tied to specific quotes was a conscious decision to mitigate identification of participants.

Inductive line-by-line hybrid open pattern sorting (a hybrid of open coding [44] and pattern data interpretation [39]) was performed (RAM) to immerse the researcher in the data, enrich the understanding of the data, and allow for free-flowing interpretation. As traditional coding can become a restrictive burden that prevents pattern recognition and impairs the ongoing narrative that is fundamental in ID approaches, hybrid open pattern sorting was chosen to embrace the rigor of traditional coding while integrating the interpretation and description mandate of ID [39]. Active interpretation and description of what the data contains was intermittently reviewed and re-envisioned being conscious of positionality and bias. Data analysis was conducted (RAM) concurrently with ongoing data collection (NM) [39]. Following a full draft of open pattern sorting, the broad list of identified patterns was reviewed by community partners. The community partners were people who work or volunteer in organizations that provide services to survivors of IPV and have substantial frontline contact with survivors. This step in data analysis was done to gain valuable insight from the additional perspectives of these individuals in the context of the research team’s interpretation of the participant’s experiences. Community partners provided further context and clarified definitions for some patterns. Community partners indicated the interpretation by the research team was thorough and representative. Two additional patterns were added to the pattern list by the community partners, finalizing the open pattern sorting stage. Once open pattern sorting was complete, the interlinking nature of the patterns and the ongoing narratives were inductively examined, re-examined, and synthesized into common patterns (RAM).

Unlike traditional qualitative methodologies, this ID approach did not subscribe to thematic saturation as a marker for study conclusion [39]. In applied healthcare disciplines, like paramedicine, an approach that fits for 99 patients may not work for the 100th. Thus, thematic saturation was not used in this iteration of the ID framework. No specific sample size was targeted in the recruitment process. The data collected was rich and did not require a certain sample size to justify value. Recruitment ceased when reasonable effort had been exerted in each of the three selected provinces to engage with survivors, and time and resource constraints led to a natural conclusion. It should be recognized that more perspectives and experiences are likely to exist but were beyond the scope of this study.

The patterns were grouped into four practical categories: (1) survivor’s IPV experiences, (2) paramedic services received, (3) challenges when receiving paramedic services, and (4) corresponding solutions for the identified challenges. The survivor’s IPV experiences was chosen as a core category to illustrate the conditions survivors of IPV were experiencing before interacting with paramedics. Evaluating this context was essential to learn about the events leading to service utilization. Paramedic services received by survivors was chosen as a core category to identify what services survivors have received. This core category also encompasses what general services survivors desire from paramedics. The paramedic services received interpretation is intentionally brief as it pertains more to context-specific instances than sweeping practices. Challenges was selected as a core category because it outlined issues survivors of IPV faced when accessing services for which solutions can then be derived. Solutions was selected as a core category to provide positive direction from the sharing of these experiences. Functional potential actionable items were co-synthesized from the lived experience as evidence from survivors and the expert knowledge of the research team. The concepts in the challenges and solutions sections were designed to be change stimulating, which was a core pragmatic value of this work built into the analysis methods. Owing to the numerous challenges faced by survivors, only the most impactful seven patterns are reported herein. This is by design to carry over an approachable number of solutions and action items from academic research to the real world. The goal was to develop evidence-based tangible action items that addressed clear challenges through linked solutions, with a goal to not overwhelm those trying to implement positive change from these results. Patterns within core categories, explanations, and exemplary quotes are presented. The rigorous design and completion of this study aligned with the Big Tent Criteria for quality qualitative research [45]. 

## Results

### Demographics

All demographic information was self-reported from the recruitment and eligibility survey and can be found in Table 1. 24 potential participants responded to the recruitment survey. Of those potential participants, six were deemed ineligible for being outside of the geographical region and nine were lost to scheduling and follow up attrition. Ultimately, nine participants participated in interviews. All participants were cisgender women. The average age of participants was 37 ± 10 years. Most participants were from a European cultural background and identified as white. Most participants were low income, and single. Employment status varied among participants. All participants experienced physical IPV. Only one participant experienced just physical IPV. All other participants experienced multiple forms of IPV.

**Table 1:** Demographic information of survivors of intimate partner violence (IPV) who participated in this research

Personal Demographics		*n*	%
Gender	Woman	9	100%
Sex	Female	9	100%
Age (mean ± standard deviation)		37 ± 10	
Cultural Background (multi-select)	African	1	11%
	European	5	56%
	Middle Eastern	1	11%
	Southeast Asian	1	11%
	Not Listed	2	22%
Race (multi-select)	Asian	1	11%
	Black	1	11%
	White	6	67%
	Not Listed	1	11%
Income Status	Low	5	56%
	Middle	2	22%
	High	1	11%
	Prefer Not To Say	1	11%
Relationship Status	Married or Domestic Partnership	2	22%
	Single	5	56%
	Widowed	1	11%
	Not Listed	1	11%
Employment Status (multi-select)	Employed	1	11%
	Unemployed	3	33%
	Student	1	11%
	Disability	3	33%
	Unpaid Carer	1	11%
	Not Listed	3	33%
Type of Violence Experienced (multi-select)	Physical	9	100%
	Emotional	8	89%
	Sexual	7	78%
	Stalking	5	56%
	Other	3	33%

### Survivor’s IPV experiences

Through the interview process, all survivors reported on their experiences of IPV that lead to paramedic service utilization. All iterations of the IPV experiences of participants yielded the common theme of partner’s intentionally exerting power and control over the survivor.Patterns can be found in Table 2.

**Table 2:** A summary of the experience patterns and exemplary quotes from survivors of intimate partner violence (IPV) about their IPV experiences preceding paramedic service utilization

Experience Pattern	Exemplary Quotes
Physical IPV	“It broke me into a million pieces, like my soul was still intact thankfully but it was detrimental to my mental health and my ability to show up in the world like I used to. I lost everything I had built for myself. Going into this, I was a competitive sponsored athlete and I did fitness modeling and promotions for health…I lost all of that. Due to all of the hits and drugging and lack of oxygen to my brain and the strangulation and X, Y, Z. I have permanent nerve damage and brain injury and nervous system dysregulations. Now I’m on disability and very limited.” “He actually dragged me. Like all my whole body to an elevator. And like the dragging my body all the way to the elevator.“
Sexual IPV	“And of having to leave work to cater to that person so I wouldn’t get the brute force of him. There was drug possession that was forced into my system. And that started leading to several rapes and being restrained by him.” “There were multiple rapes between all of this timeline, forced objects.”
Psychological IPV	“He then proceeded for two days while I was at work to message me non-stop, threatening that he was going to hurt or beat up or confront literally any male that has ever talked to me in my lifetime.” “You’re worried about the next breath they take. It sounds different. Like, oh no, should I be afraid? That’s not right. That’s not love. It’s not partnership.” “And my dog jumped on the bed to protect them, and he put the knife to his throat and said that he was going to start with my dog.” “And then I got so debilitated by the fear that I remember just laying on the ground shaking.”
Coercive Control IPV	“I’m not allowed to say on anything, and he took my cell phone away from me, take all my ID away from me. And with my bare feet and he just kicked me out from his car in the middle of somewhere.” “He got into my building where I was living and was trying to force his way into my unit and beg for me back.”
Daily Survival	“I’m just, I’m always scared. I never ever feel safe anymore.” “Cause even after I’d kicked him out, he was still stalking and harassing me so I ended up taking a bunch of pills and drugs and alcohol to try and induce and escape.” “I just put up with all this violence from my partner at all times.”
Cycle of Violence	“He moved in with me that, after that reuniting, and that’s when I got physically assaulted.” “For 8 months and most of the relationship was emotionally abusive before it was physically abusive.” “It was a very like slow, like he was super awesome and charming and amazing for the first year. Like and I guess the first ten to eleven months and things start to start slowly getting worse, and then the last six months were just absolute warzone.”
Partner Factors	“I don’t know it’s messed up because you love that person, so you don’t want to get them in trouble.“ “Another big reason why I never reported anything with any partner was due to the abuser gaslighting me and making everything my fault.” “He was running out of his money. He was quite upset.” “And he apologized to me. While he did not remember what he asked or did to me and because he was so drunk that he just cry and then “*I’m so sorry*,* I don’t know why do I do? And I don’t remember what I told Police about. I*,* I hit you*,* right?*”. And that’s what he said.”
Survivor Factors	“And women living in such a state of fear constantly and who are scared to ask for help because what happened last time they asked for help or someone called for help for them on their behalf. Again, even if people are doing the right thing, they don’t know the repercussion that happened to that woman or male or they because of that call and putting attention on that person.” “Somewhere back in my head, I have to protect him. And if I tell the truth, he’s gonna be in a jail.”

Participants expressed they had experienced a wide range of physical IPV assaults. Direct blows (e.g., punches, kicks, slaps), strangulation (e.g., hands around their neck, objects around their neck), being assaulted with weapons (e.g., bats, knives, hammers), and being thrown down stairs or into walls were common. Without prompting, participants reported signs and symptoms consistent with IPV-caused brain injuries (e.g., headaches, trouble focusing, balance issues, low distress tolerance) following physical assault. When explicitly asked, participants reported further signs and symptomology consistent with IPV-caused brain injuries (e.g., confusion, loss of consciousness, heightened sensory sensitivity). Iterations of these signs and symptoms were reported after common mechanisms (direct blows, indirect blows, etc.) and non-fatal strangulation. Fractures, craniofacial trauma, soft tissue injuries, and loss of consciousness were regular results of physical IPV. In severe cases participants reported being drugged, being assaulted resulting cardiac arrest, kidnapping, entrapment, home invasion, and having vehicles used as weapons against them (e.g., being run over, having the perpetrator cause a crash to cause harm). Participants reported that contact orders or bail conditions were not useful in ensuring their safety, as perpetrators were not deterred by these documents.

Participants reported that sexual IPV was a regular occurrence. Sexual IPV experiences disclosed by participants included rape, forced penetration with various objects, forced sex work, date rape, and human trafficking. For safety reasons, investigative questions about sexual IPV were not asked and specific disclosures were only made when participants wanted to share their experience in greater detail.

Psychological IPV spanned many techniques weaponized by perpetrators. These techniques included verbal abuse, threats against participants, their children and their pets, intimidation, instilled fear, violence in public to demonstrate that no one cared and the perpetrator could do as they please, embarrassment, gas lighting, victim blaming, and manipulation. Participants indicated that detrimental psychological state changes were common, further compounding the negative aspects. Mental health disorders emerged and/or were exacerbated among participants living in situations of chronic abuse. In cases where participants belonged to marginalized groups, culture shaming and racism were also uniquely employed as psychological IPV.

Coercive control spans any efforts used to intentionally exert power and control that are not covered under the definition of physical, sexual, or psychological IPV. Numerous violent behaviours were experienced by participants including stalking, forced social isolation, financial control, restricted cell phone and internet access, tracking participants movements, stalking members of the participants social group, cultural isolation, and promises of change.

For participants, the sense of normalcy of a violence free life quickly evolved into the sense of normalcy of a life with IPV. In order to function in that environment, participants reported using different approaches to survive. Participants aimed to avoid their partner’s violence triggers (e.g., not having dinner ready, not keeping the house clean), normalized the violence as a perceived reality, and hoped for change. Police were reported to commonly interact with participants and perpetrators of IPV. These interactions yielded positive effects like the perpetrator’s arrest and de-escalation, but also resulted in heightened IPV upon the perpetrators release, disbelief of the survivor when disclosing to police causing increased violence later, and a distrust in the process and system.

The cycle of violence and contributing factors were described by participants. Participants reported IPV often started following cohabitation and that once violence had begun it was cyclic and escalating. There became immediate concern for the participants safety, specifically when attempting to leave violent relationships. Direct threats (e.g., “*I’ll kill you if you leave*,* I’ll find you and I’ll kill you”*) were used to control the participants and instill fear. In addition, participants reported various levels of dependency on their partners for housing and finances. As participants felt socially isolated with their violent partners following cohabitation, limited social support resources were available, and the violence continued. Honeymoon periods of apologies and gifts occurred, but violence persistently returned.

Among perpetrators, participants reported some shared features. Perpetrators were described as extremely manipulative. Perpetrators would tell their partners they have no option but to stay with them, the repercussions if they left would be dire, and that no one cares about them. Some participants reported their abusive partners had preexisting mental health and addictions challenges, and some reported there were no flags, mental health or otherwise.

Childhood trauma was a common experience among most participants, with a family history of IPV, physical child abuse, and sexual child abuse being prevalent. Most participants also reported polyvicitmization across multiple relationships. Participants who reported polyvictimization possessed a constellation of brain injury signs and symptomology. Belonging to a marginalized group (e.g., visible minority, low socioeconomic status, etc.), and having low self-esteem were typically shared participant characteristics. Participants reported they played a role as carer for the perpetrator, whether for their rehabilitation from perpetrating violence or preexisting mental or physical health conditions. Substance use to cope with violence was reported, but not frequently. Suicide or self-harm were considered by multiple participants as ways to cope or escape the situation, with a corresponding history of suicide attempts for some participants. Although some participants reported preexisting mental health condition prior to experience IPV, all participants described impaired mental health and/or novel mental health diagnoses after being exposed to violence.

### Paramedic services received by survivors

Participants reported on their experiences using paramedic services. Patterns can be found in Table 3.

**Table 3:** A summary of the experience patterns and exemplary quotes from survivors of intimate partner violence (IPV) about their experiences with paramedic services

Experience Pattern	Exemplary Quotes
Paramedic Service Access and Utilization	“Most of the times it’s been people from the transition house after something happened. And one time I had to call myself, just with like the SOS button on my phone.” “And it turns out that he had roofied me and drugged me to the point of my heart stopping in my sleep and I was blue, not breathing, and [he] didn’t want charges of murder, so he called 911.”
Services and Treatment Received from Paramedics	“To my knowledge, the major event of me going into cardiac arrest, [the Paramedics] were very prompt, very quick, and good… they revived me, and it took me a bit to come back to the living world, but, I came back, so that was thanks to the paramedics.” “This incident, it’s like at the end of too many times. I opened a door and there’s like seven plus EMS, and first responders, and police, and all these different levels of [Responders] I’ve never seen in my whole life.” “So, they call the police for me. And then the paramedics came in and checked on me, just checked some vitals. [The Perpetrator] didn’t tell the truth [to the Police]. The police didn’t come back to me and ask me all the questions. Police didn’t take into the serious consideration of what happened.”
Approaches and Interactional Factors of Paramedic Service Use	“So, I just. I just broke, and then they only checked my pulse. “*Your pulse is fine.*” And so, “*can you see things ok*? *Can you hear me okay?*” That’s all they tested. And I’m like, “*yup*.” And [they said] that it’s nothing priority…I wish there was more support and follow up after that incident.” “That’s it, yeah. Crisis intervention. Yeah, like the trauma-informed practice. That’s the word. We’re not just gonna label you as one thing, like, “*oh yeah*,* you’re with the guy who beat you up*,” like why are we labeling people? Like, let’s us understand that there’s so much more…Like, let’s understand that there’s so much more to a story and you are the first responders to somebody. It was, yeah, shocking really. I don’t even know what to say about it.” “It’d be nice if they would suggest me to go to see a counsellor, for example.”

When considering services received, participants reported that they would call 911 for police and/or paramedics in severe circumstances and if they were able. Third party callers (e.g., neighbors, children, etc.) would also call 911 when suspicious of episodes of IPV. Depending on the situation, participants would call for emergency services from a location different to their home. Sometimes this was to disguise the IPV situation or was following fleeing the house. All participants had called 911 for IPV, but this was primarily done in severe instances. Less severe instances did not frequently result in a 911 call from the participants.

According to participants, when paramedics attended, they assessed for and managed injuries. Paramedics often co-responded with police. Paramedics would typically interact with the survivor and the police would interact with the perpetrator. Frustrations amongst the paramedic crews when they attended the same situation repeatedly were reported by the participants. Outside of the basic patient assessment, no unique or specific approaches were reported that account for the complexities of situations involving IPV as a clinical circumstance.

The approaches paramedics used varied from crew to crew. Some crews were reported to have been respectful and try to meet the patient’s needs. Other crews were reported to have been discriminatory and rude. Participants reported they were hoping to receive empathetic, trauma-informed, and effective care from paramedics. Physical injury care was only one portion of what participants hoped paramedics could do. Participants stated they wanted versatile options to access healthcare and community services, beyond just a ride to the emergency department. Participants hoped that paramedics could link them to appropriate services and be a stepping stone towards positive change in their lives. This was not always mirrored by the desire to exit a relationship, but rather to be in non-violent situations, encompassing exiting the relationship or improving the relationship. The desire for holistic service access that could be applied to the participants stage of their life and relationship while accounting for their overall circumstances was repeatedly echoed.

### Survivor’s challenges to receiving paramedic services

The seven most impactful challenge patterns experienced by participants as they accessed and used paramedic services were bias and discrimination, individual paramedic conduct, undereducated and untrained paramedics, insufficient paramedic service infrastructure, transitions into healthcare and community services, perpetrators factors, and patient factors (Table 4). These challenges spanned societal, perpetrator, and participant derived challenges, but focused primarily on adversities specific to paramedics and paramedic services.

**Table 4:** Challenge patterns and exemplary quotes from participants who are survivors of intimate partner violence (IPV) who were attended to by paramedics for IPV-caused reasons

Challenge Pattern	Exemplary Quotes
Bias and Discrimination	“No one came to talk to me and I feel that is because of I’m a minority.” “I felt more scared and I wanted to get out of the vehicle and I was saying just kidding don’t take me to the hospital.” “[Paramedics] is not, should not be projecting out to anybody. Like it doesn’t make sense to do that to somebody who’s in a crisis.”
Individual Paramedic Conduct	“But if I was, like, alone or someone else had called and I was in the place where I was alone, it was, it felt very dehumanizing, like, the way I was, like, being spoken to and, like, I’m 23 but I know, like, I look a little bit younger and my voice gets, like, really high when I’m stressed so just, like, constant comments about, like, I’m in high school I should go home, like, this wouldn’t be happening to me if I was just at home and I’m like I am a full adult. I can’t go home actually. So yeah, just a lot of unsolicited opinions.” “[The Paramedic] said, “*well*,* maybe this will teach ya not to go back this time.*”” “But the ones in [Province] were not great in regards to being trauma-informed and de-escalating the situation…I felt like those [Other Paramedics] escalated and made my state way worse than it needs to be.” “There was a time where it was just like, they were so insistent and like kept getting closer to me and like putting their hand on my leg because they really wanted to look, and I just almost kicked him. It was usually an okay experience if it was a woman.”
Undereducated and Undertrained Paramedics	“I didn’t feel like I was properly cared for. I felt like I was more, I got more terrified with the paramedic team.” “There was one time where it felt very physically invasive as well, but mostly it’s just like the lack of trauma-informedness around language.”
Insufficient Paramedic Service Infrastructure	“I reached out for help. It was not held in confidence and it was manipulated and contorted and put me at more heightened risk.” “I do think there are some loopholes in regards to domestic situations, follow up and follow through.”
Transitions into Healthcare and Community Services	“I’m not sure if its protocol to just drop them and leave but that’s what happened.” “Do they talk well together? No. Do we know how to find them? Okay. Well, no. Not very well.” “I know that their intention was to get me somewhere safer and make the hospital basically do their job, because they were doing theirs. So, I think on that end, that was the hospital’s fault, not the paramedic’s fault.” “Paramedics did save my life. But, I wasn’t protected from it ever happening again. I was served on the platter back to the abuser.”
Perpetrators	“I know that that I’m the one who should be telling what happened, what he actually did it to me, but it’s so hard for me to tell that to in front of my partner.” “During the second incident, my partner and I sat down with EMS all together in our apartment. I did not have the courage to discuss what happened. I had an anxiety to talk about intimate violence with EMS especially when my partner was sitting together.” “My partner can be very manipulative and when I got handcuffed. We are not allowed to contact each other…I was in shock that he was actually telling a lie to our neighbor and said I was in mental health episode.”
Patients	“Another big reason why I never reported anything with any partner was due to the abuser gaslighting me and making everything my fault. As well as the embarrassment associated with being in that type of relationship.” “A lot of that I think, stems from the lack of oxygen to the brain and the multiple hits to the head and then the forced drug use.” “I thought I was gonna have my eyes ripped out. It started with that cause I was trying to leave and he wanted to fix our problems right now by grabbing my face from digging his fingers into it. I feel I have scars in my eyes there from his nails. And that’s where it starts.” “Well, I have complex PTSD. It’s really hard. I don’t…I can’t trust people. I feel like I’m just very like fragile.”

#### Bias and discrimination

When attempting to access paramedic services, participants reported that various factors often contributed to them being subject to bias and discrimination. Race, immigration status, being culturally and linguistically diverse, and not being fluent in the medium of English were factors that increased the risk of marginalization among participants. Homelessness, housing insecurity, and low-income housing were reported as predisposing services providers to hold negative connotations towards the participants to which they were supposed to be providing services. This bias persisted into attendances at emergency shelters. Noting that housing insecurities were common among those exiting or attempting to exit violent relationships, this discrimination was reported to push some survivors to return to violent homes where they cohabitated with the perpetrator. The culture of the participant and/or the culture of the perpetrator were also believed to impact the threshold for bias and discrimination. In general, problematic social determinants of health appeared to have situated participants at risk of marginalization. Evidence of substance use (e.g., alcohol, opioids) at the location paramedics attended, regardless of who was using the substances, would commonly lead to adversity for participants. Being belittled, not being believed, being labelled, and being dehumanized were common outcomes of discrimination.

#### Individual paramedic conduct

Some participants did report positive interactions with individual paramedics. However, the vast majority of incidences were reported to be negative. Participants stated that paramedics would impose their personal beliefs and interpretation of a situation onto the participant and act accordingly. These behaviours included shaming and blaming the participant, criticizing the participant for not exiting the relationship, using the participants cultural context to justify IPV, and informing the participants that if they did not act to change the situation it was their fault, and they *deserved* to be abused. Participants reported they felt disrespected, not heard, not seen, and not believed. The interactions with individual paramedics were reported to be harmful, as opposed to beneficial, and encompassed an array of negative experiences with paramedics. These negative experiences were reported to repeat and worsen if paramedics attended repeatedly, as was common for participants experiencing cyclic violence. Detrimentally as cyclic violence was commonly also escalating, participants reported receiving worse care from paramedics as their needs grew and safety risk worsened visit after visit. The overall attitudes of providers who were attending the participants were reported to be generally negative towards survivors of IPV. Participants felt paramedics exhibited signs of disinterest and lack of engagement that participants felt would not have been comparable if they were a standard medical patient. Multiple participants reported they felt they were at risk of homicide at the hands of their intimate partner, but that the paramedics would not take them seriously. After repeat attendances by paramedics in a situation of cyclic and escalating violence, one participant experienced an IPV-caused asphyxiated cardiac arrest. Fortunately, they were successfully resuscitated. Cumulatively, the participants reported the personal beliefs and interpretations of individual paramedics impacted their care and were unfavourable towards survivors of IPV.

#### Undereducated and undertrained paramedics

The undereducation and undertraining of paramedics was consistently referred to as a challenge experienced by participants when using paramedic services. Participants first referred to a lack of IPV recognition by paramedics. Many reported that despite obvious signs and symptoms, they were never questioned about IPV or asked follow up questions if a situation was known to paramedics. When participants did disclose IPV to paramedics, it was reported that paramedics frequently did not know what to say or would impose their personal opinions. The response from paramedics was reported to lack empathy and direction. Participants specifically reported a distinct lack of a trauma-informed response by paramedics. Poor communication, poor psychological care, and absent safety considerations were noted by participants. Further, participants reported that their physiological response to trauma often impaired their ability to respond to questions appropriately, which led to distain from paramedics. The assessments paramedics conducted were reported to be either basic and minimal (e.g., partial vital signs), or extremely invasive and unreasonable (e.g., examining genitals without cause). Participants reported being given minimal options by paramedics beyond being taken to the hospital. Participants stated that paramedics did not have knowledge of community resources and were not sure what they could do for patients beyond transport to an emergency department. A few participants gained access to their healthcare records and reported paramedics were doubtful and possibly accusatory (e.g., *patient’s story was inconsistent with scene findings*), extremely basic, and had no mention of IPV. Participants reported that there was no special consideration given in situations of IPV and that many gaps existed in the care they received around the clinical circumstance. Participants explicitly reported they felt paramedics were not trained to manage cases of IPV and did not appear functionally ready.

#### Insufficient paramedic service infrastructure

From a paramedic service infrastructure standpoint, participants reported many issues. Participants reported that following a paramedic attendance that nothing changed. Even in situations when participants were taken to hospital, there was no tangible benefit. Participants reported being transported to emergency departments. The options participants were provided were transport to an emergency department or staying where they were. The emergency departments were reported to feel inappropriate as they lacked available resources (e.g., no social workers, no counselling, no safety planning) to support participants, and felt unsafe, especially when triaged to the public general waiting room (accessible to perpetrators). Participants reported it felt like there was no process to move through and that the help they required was not available to them through paramedics. Factors like children, pets, and safety meant that transport to the emergency department was not always feasible. In these cases, the only other option was staying situated, frequently at home. When left at home, participants reported their safety was at risk and they feared repeat violence or homicide. Participants reported absent follow up, absent referral, and no trust in the process. Further, participants reported that a definitive disclosure was presented as the only way to access resources, and that even after a forced disclosure it still seemed that nothing happened. The lack of knowledgeable specialist teams was identified as a challenge. Participants reported they were predominantly attended to by paramedics who were men. Considering participants had just been assaulted by men, they indicated there was a component of fear and distrust when being attended to by paramedics who were men, especially if the interaction was a negative experience. It was also reported that when paramedic records were used in legal proceedings, that there were never reports on the perpetrator. Participants felt like there was a missed opportunity to gather evidence (e.g., paramedic records documenting bruised knuckles and cuts on the perpetrators hands after a physical assault that could corroborate the participant account) based on the physical findings of the perpetrator. In line with insufficient records, participants quickly learned that paramedics could not see the reports from previous paramedic attendances. This led participants to have to repeatedly tell the attending paramedics their story. This was traumatic reliving violence, frustrating considering nothing changed, and dangerous because it put participants at risk of the perpetrator’s wrath if they became privy to the reporting of IPV. Participants also reported that their addresses were flagged in police systems, but not in paramedic service systems. So, if paramedics attended without police, then the paramedics could also be at risk of violence from the perpetrator. The paramedic services infrastructure, or lack thereof, left survivors feeling underserviced and vulnerable. Participants felt that the people who were supposed to provide them with out-of-hospital healthcare support (paramedics) were not positioned to do so, and they were affected with the consequences.

#### Transitions into healthcare and community services

Participants believed that while paramedics were not the ultimate resource for survivors of IPV (e.g., not expecting counselling or housing support from paramedics), a primary function of paramedics should be the transition to services to address their holistic health and wellness needs. However, many issues were identified within the transition to healthcare and community services phase of paramedic care. The disjointed nature of transition to other services was the primary and common challenge in this category. The primary pathway paramedics provided for participants– transport to a hospital emergency department– did not commonly yield access to services for participants. Participants who were brought to the emergency department by paramedics reported that the hospital staff they talked to were not made aware of their IPV situations, leaving participants to be re-traumatized by having to brief yet another healthcare provider. This also led to major delays in meeting with social workers or case workers, as referrals had often not been initiated. At smaller centers, social workers were not scheduled at night and participants were discharged before ever seeing one. Participants who were deemed to have suffered no injuries or minor injuries were frequently moved to general waiting rooms, where they waited for hours. Participants reported being scared their violent partners would enter the waiting rooms and attack them. Many participants were discharged from the emergency department without being provided access to specialized resources and were advised, if at all, to follow up with community services independently. Participants reported frequently that they did not know how to access community resources or know what resources were available to them. In situations of non-conveyance, participants reported receiving no follow up or referral. In situations where police also attended, participants reported occasionally being given a phone number for victim services. Participants reported physical cards were not always safe to hold on to as their partner may find it and respond violently. Interactions with police were often void of follow up and relied on direct disclosures to access resources. Participants reported that unless someone followed up with them, they often would not seek out specialized services. They reported often forgetting to contact services, not having transportation to services, or being concerned of the repercussions from their partner of seeking services. Crisis lines were often useful in acute crisis but would commonly result in a recommendation to call 911 and being attended to by paramedics and/or police. Participants reported repeatedly falling through the cracks and having the buck passed along to the next service or group, which was ultimately ineffective and resulted in the participants being underserviced. Participants reported losing trust in the system and being less likely to call 911 or access the emergency department because of the inaction on previous attempts. The transitions into the healthcare system also exposed participants to similar bias and discrimination, personal belief patterns, and process deficiencies as paramedic services. Participants also report a lack of autonomy in their healthcare decisions. One participant reported being discharged into the care of their abuser, which became violent soon after returning home. The transition from paramedic service to healthcare and community services that met the participant’s needs did not occur for any of the participants, despite repeated interactions with paramedics over multiple touch points.

#### Perpetrators

Participants reported challenges with their partners when being attended to by paramedics. Participants reported that their partners would not typically be aggressive or violent towards paramedics, but rather use deceit and manipulation to influence the attending first responders. In situations where perpetrators were interacting with emergency services members, it was described that they would be able to fraternize so as not to implicate themselves. This technique would simultaneously discredit their partners claims of violence. Perpetrators were also reported to discredit the participants by claiming they were drunk, high, or crazy. Perpetrators were reported to have different triggers, like mental health concerns and addictions, that could cause bias when paramedics were attending the scene that extended to the participants. Perpetrators would commonly restrict or deny participants access to phones or other means to contact help. On scene when paramedics were in attendance, participants reported perpetrators would linger and prevent participants from speaking freely with paramedics.

#### Patients

The participants identified that they themselves could be challenging for paramedics. Especially when they had been attended to previously by paramedics and had negative interactions, the participants reported they did not want to engage for fear of another negative experience. Participants reported that having children in the house they did not want to leave with the perpetrator or could not leave unattended would cause them to chose not to attend the emergency department. Participants reported lacking social support and feeling isolated, which compounded their opposition to attend the emergency department and to leave their relationships. Participants reported they had an unexplained love for their violent partner and a hope it would get better if they did not expose the situation. Survivors reported feeling perplexed about these emotions but stated they were real feelings that they could not otherwise explain. Many had indicated they had accepted the situation and there seemed to be a cultural acceptance of violence towards them. Participants were adamant that they did not trust the process would be effective and felt it could not guarantee their safety. As accessing services and disclosing IPV was likely to trigger violent episodes from their partners, without guaranteed safety they felt the most survivable option was to remain and not comply with paramedic advice. Participants reported supplying deliberate misinformation and false reporting to paramedics. The stigma around IPV, the feeling of embarrassment, and the feeling of shame also resided with participants as a reason for not wanting to disclose IPV or engage with paramedics. Participants reported simply trying to survive everyday in a world of violence. When they were isolated, injured, downtrodden, and saw no hope, the drive to engage with service providers was often absent. When paramedics would engage in a way that exacerbated those preconceptions, the spiral would often continue for participants. Outside of paramedic services, participants often had no primary healthcare access and held a general distrust of healthcare providers.

### Survivor’s solutions and actionable steps

In response to the seven most impactful challenges reported by participants, a corresponding list of participant-derived solutions paired with functional potential action items to progress towards those solutions co-developed with the research team are found in Table 5. There was considerable overlap between actionable items at different levels of change. This highlights the importance of concurrent advancements in multiple areas and the inter-related nature of these challenges and solutions.

**Table 5:** Participant-identified challenges and corresponding participant-derived solutions with exemplary quotes to highlight solutions and potential actionable items to enact change towards those solutions for situations involving intimate partner violence (IPV)

Challenge Pattern	Solutions	Exemplary Quotes	Potential Actionable Items
Bias and Discrimination	Safe and Equitable Paramedic Cultural Positioning	“They were fantastic, and they helped me come back into myself and they removed me from the unsafe.” “Let’s get that first initial, like, “*you are safe*” and keep on encouraging that because then the person that’s in crisis would be, you know, it’d be easier for them to receive the services too. They’re not going to be objective or argumentative or, you know, push back or whatever.”	• Build and enforce zero-tolerance policies towards bias and discrimination • Prioritize creating a safe environment with credible rapport between the patient and paramedic • Increase resources to reduce workloads and increase capacity for empathetic care • Emphasize a culture of patient centred-holistic care
Individual Paramedic Conduct	Respectful and Professional Conduct	“Yeah, so some of the positives were how they brought light to situations. They, some of them seemed very caring and very attentive and respectful of not only me and my body in space, but the circumstance and environment. They were gentle, with moving me from one place to another. They were, consoling, like, made me feel safe and calm after being in a very unsafe and uncomfortable state and situation.” “They did get me out of the unsafe environment, and they called me by my real name and we got to the hospital, and so I appreciated that.”	• Avoid accusatory language in conversation and documentation • Provide clear communication while being mindful of language barriers and the response to trauma • Reflect on and mitigate personal biases • Respect patients, treat all patients equally, and avoid stereotyping or assumptions • Validate the survivors experience with empathetic and supportive care, and refrain from blaming or shaming • Foster a professional and compassionate attitude
Undereducated and Undertrained Paramedics	Mandatory and Ongoing Education and Training	“I think it’s very important that they understand the effects of trauma or crisis like that.” “There’s a lot, like I’m very grateful that I’m here today and it’s because of paramedics and I’m very grateful. But lots has come up around frustration. Looking back and going through all this with you and the questions made me realize how many times I could have been saved and removed from that situation versus having to get that. And if there was more education, I wouldn’t have been so ignored.” “I do think empathy was there and, being trauma-informed. Last year when I had the paramedic experiences in [Province], they were great.” “I was gonna say they were really good at deflecting the bad and focusing on getting me, to focus on the patient’s needs versus the environment around and what was being said.” “And I really do think that they would benefit from more education and trauma-informed practice as they say nowadays become trauma-informed but also you know how to properly assess the situation.”	• Educate paramedics about IPV, how to recognize IPV, how IPV-caused brain injury and non-fatal strangulation can impact patients, and how paramedics can support survivors • Train paramedics to adopt a trauma-informed care approach that prioritizes safety, trustworthiness and transparency, collaboration, empowerment, humility, and responsiveness • Educate paramedics on available resources, pathways, and options to meet patient needs • Train paramedics to identify options to best meet patient needs (e.g., transport destinations, referral services) • Educate and train paramedics on how social determinants of health can impact care, and strategies to mitigate these factors • Equip paramedics to collaboratively build a care plan with survivors of IPV that highlights all their options, will meet their needs, and emphasizes patient autonomy
Insufficient Paramedic Service Infrastructure	Develop Functional Paramedic Service Infrastructure	“Police involvement, social work involvement. Be transported not only to hospitals but maybe get Police or RCMP involvement and get that get the domestic person, whether it’s male or female, somewhere safe.” “Little things like, you know, maybe put some, after care suggestions would be something that just adds to feeling and experience, and take that home.” “And coordination between services and agencies. Like law enforcement should absolutely be involved, social services, shelter, nurse, psychologist, all to address intimate partner of violence effectively.” “There’s so much that is thrown out. They could say it all perfectly, but how much is going to get retained in those moments?”	• From dispatch onwards, develop the ability to link and review multiple contacts with paramedic services for situations involving IPV • Build uniform recognition and screening tools into paramedic documentation software as a point of care resource that lowers paramedic burden and automatically advances potential survivors of IPV to appropriate resources • Develop and integrate clear and versatile pathways to holistically meet patient needs through seamless facilitated referrals and transitions to healthcare and community services with minimal burden on paramedics • Integrate considerations for real world constraints (e.g., children, housing, cost) into continuum of care pathways • Strengthen interdisciplinary collaborations to allow two-way communication among attending agencies (e.g., police, social work, community care) to establish a unified plan for each patient • Provide closed-loop communication to paramedics to reinforce their actions facilitated the appropriate response • Create policy that supports the functional development of the corresponding infrastructure
Transitions into Healthcare and Community Services	Develop Functional Transitional and Collaborative Infrastructure	“They’re like, okay, just gonna go back. Just easier. And then it’s just the state, you know, it’s that vicious cycle of abuse. So if there’s, you know, coordination between all service providers to ensure somebody actually can stay away is super important.” “I think if that was, if that grew into a more, I guess, integrated relationship and approach to arriving on scenes and to calls. I think there’d be a lot less repetitive domestic abuse situations to the point where for me it put me in a cardiac arrest and almost lost my life.“ “If we can have like a wrap around service where, you know, the initial response with the police then the paramedics to the hospital, the hospital calls out, you know, the shelters, the shelter obviously is a temporary solution, like, where are we going to put them next, like, where are they safe, like, what are they gonna do during the day, like, there’s all these things because the second that the person is left alone or feels vulnerable, I feel like they’re so easy to return.” “So I think that is very important that the community providers, especially frontline workers and emergency services, sit together to say, “*hey*,* I have this available for if something comes up.”*” “Not leaving patients, I guess, alone after. You are giving them back to, are putting them back into an abusive situation or on healthy situation.”	• Collaborate with fellow component groups (e.g., emergency departments, emergency shelters, community services) of healthcare and community service pathways to establish clear processes with functional continuums of care • Develop pathways for situations of hospital conveyance (e.g., transport to the emergency department or urgent care), non-hospital conveyance (e.g., emergency shelters), and non-conveyance (e.g., survivors remaining in community), all with built-in follow up • Automate process initiations (e.g., when potential IPV is flagged in ambulance documentation, then an automated trigger enables the continuum of care process with the next provider in line) among pathway component groups • Build interdisciplinary response networks to coordinate care and reduce transition gaps in real-time • Standardize pathways, ensuring all relevant information is advanced into healthcare and community services groups • Adopt a universal education approach, where a disclosure of IPV is not a barrier to receiving information and referral options
Perpetrators	Integrate Techniques to Address Partner Impact	“The first incident happened, and the paramedic came in and talked and then asked me to get into the to ambulance the to check on me and they probably asked me what happened and I say “*he hit my head or punch my head*”…he was away from me. He was with the police.” “Separating during assessment and talking to you.” “I think something that could be really useful and things I have heard from other people is just more training on being able to like recognize the signs of when there has been, like, violence. When the two people are in the same room and being able to recognize or at least have enough to like make a suspicion and then remove the other person, very calmly remove them in a way that wouldn’t make them angry and get them to like retaliate against the person who at was a victim of the violence.”	• Utilize tact and situational awareness to occupy and remove the partner to allow for private interactions with paramedics • Be conscious of the gender of the paramedic having the private conversation with the survivor, as an isolated interaction could trigger the perpetrators violence • Collaborate with other responders (e.g., police) to manage on scene dynamics • Attempt to remain objective and avoid being deceived or manipulated
Patients	Integrate Techniques to Facilitate Patient Benefit	“I was calmed enough for me to move on with things because I was given an opportunity to speak. So, I was heard and that was enough for me.” “We’re not just gonna label you as one thing like, oh yeah, you’re with the guy who beat you up, like, well, you know, like, why are we labeling people? Like, let’s understand that there’s so much more to that to story.” “I just want to be like respected and also have them, like, respect my autonomy as well.”	• Validate the survivors experience and inform them there is a process intended to support survivors • Build rapport and use good communication techniques to mitigate concerns of embarrassment and stigma • Develop plans to meet survivors where they are at, highlighting functional basic safety planning • Inform survivors of their options and reinforce that they have autonomy over their pathway

Participants reported that mitigating the corresponding challenges would have been beneficial when they were accessing paramedic services for IPV-caused reasons. Potential actionable items were developed with participants where possible, and otherwise in the context of the participants responses with the expert knowledge of the intersection of paramedicine and IPV by the research team.

## Discussion

This study examined how the perspectives and experiences of survivors of IPV who have been attended to by paramedics may inform the understanding of current clinical practice and guide potential improvements for paramedicine. This research provides evidence generated from the lived experience of survivors of IPV who were attended to by paramedics for IPV-caused reasons. This distinction is crucial because there is a lack of evidence highlighting paramedics are attending calls for IPV and providing services to survivors of IPV. By highlighting the types of violence and circumstances participants experienced, insight was gained into the clinical circumstances survivors may face when they became patients. The descriptions, although preliminary, of the services received from paramedics by participants document the first insight into the clinical practices of paramedics when encountering survivors of IPV. The disparity between the services received and what participants desired from paramedic services illuminates and supports with data the likelihood that survivors of IPV are being underserviced by paramedics and paramedic services. As this work was designed to be change stimulating, the participant derived solutions paired with tangible action items can provide pilot guidance for paramedics and paramedic services looking to advance their care for survivors of IPV.

Although specific challenges may exist in the context of paramedicine, survivors of IPV are known to experience challenges when accessing healthcare and in-community services [46–49]. It is likely that the bias, discrimination, and ongoing challenges faced by survivors could persist during their transition into the healthcare and community service systems. While obvious challenges faced by survivors of IPV when accessing paramedic services need addressing, widespread systemic issues persist beyond the scope of paramedicine alone.

To our knowledge this is the first study to exam the intersection of paramedicine and IPV from the patient perspective. Leppäkoski et al. engaged with survivors of IPV in the emergency department using a questionnaire to find out about their experiences, barriers, and facilitators when accessing services in-hospital [50]. This research adds to the field in a manner beyond Leppäkoski et al.’s work by highlighting that barriers to receiving services are occurring before survivors of IPV ever reach the hospital, and that survivors of IPV frequently do not make it into the hospital at all. A disconnect between in-community services and in-hospital healthcare services may exist. To further incorporate the community-based perspective of survivors of IPV who may not be accessing the healthcare system, future work should continue to learn from the perspectives of non-healthcare, community-based IPV service providers in different contexts, including paramedicine.

The participant’s experiences of IPV were horrific. The extent to which survivors of IPV were exposed to violent acts was shocking and unacceptable. Leaving a violent relationship can be lethal for people experiencing IPV [33, 51]. Heightened IPV, threat of homicide, and attempted homicide were reported when participants were exiting violent relationships. This is in alignment with findings that the risk of homicide is increased when exiting a violent relationship [33]. The misinformed “*why don’t they just leave*” argument neglects this risk. For paramedics, being cognizant of if a survivor is attempting to exit a relationship, and how the corresponding level of violence may have fluctuated are essential components of understanding the risk of harm for the patient to guide clinical decisions.

The commonly reported finding of cyclic and escalating violence [1] was mirrored among participants. Participants routinely experienced repeated violence by their partner and polyvictimization by multiple partners. This is of particular concern when considering the impact of IPV-caused brain injury. IPV-caused brain injury is quickly becoming established as detrimental component of IPV [22]. Non-fatal strangulation was prevalent and is another mechanism that contributes to IPV-caused brain injury [52]. Repeated violence and polyvictimization in the context IPV-caused brain injury would suggest that survivors of IPV who are being seen by paramedics are likely to, knowingly or unknowingly, have endured one or more traumatic brain injuries. As enduring a traumatic brain injury increases susceptibility [20, 53–55], repeated brain injuries with minimal clinical care or recovery are likely. Paramedics and paramedic services should build considerations for IPV-caused brain injury into practice at the intersection of paramedicine and IPV. Practice changes could include IPV-specific brain injury screening assessments or incorporating presumed brain injury care as standard practice for survivors of physical IPV.

Among participants, 911 calls typically originated in instances of severe (versus their normal) IPV. For example, survivors of IPV may be physically assaulted with punches, slaps, and kicks on a daily basis, but a relatively severe incident would be one that has progressed beyond that with increased force and volume or use of other techniques. Given that participant experiences were indicative of cyclic and escalating IPV, additional patient safety and injury considerations should be exercised by paramedics when attending IPV calls.

Paramedics and paramedic services must consider the exposure to violence against paramedics in volatile calls involving IPV. Violence against paramedics is a chronic problem, frequently resulting in injury and time away from work [56]. 

When violence is unidirectional and no injuries are reported by the perpetrator, it is unlikely paramedics would conduct an assessment on the perpetrator. Participants indicated this could be a missed evidence gathering opportunity to support neutral fact finding. An ambulance care record or report on the perpetrator could highlight physical evidence (e.g., bruised knuckles, scratch marks from self-defence injuries) that could corroborate a survivor’s story (e.g., being punched, scratching the perpetrators arms to stop strangulation). When feasible, consideration should be given to assessing and documenting findings on both the perpetrator and survivor.

Participants reported a vast array of challenges that may represent generally common experiences for many survivors of IPV who are being attended to by paramedics. Lack of support for survivors of IPV can contribute to compounding and worsening violence, and homicide. The evidence reported in this research showcases that many challenges exist, and that there are opportunities to do better.

Given the substantial challenges faced by participants when accessing and using paramedics services, the time to mobilize positive change is now. As paramedic practice and paramedic service evolve into a more evidence-based era, the versatility of paramedics makes now an exciting time to be involved in the advancement of the profession. Novel pathways for different clinical circumstances are being developed and deployed to better meet the needs of patients [57]. Parallel options may be beneficial in supporting survivors of IPV.

The functionality of any advancements must be considered. In Mausz et al.’s recent work exploring workplace violence against paramedics, the implementation of functional and automated-referral violence reporting with timely closed-loop feedback found astounding rates of violence against paramedics [56]. It is unlikely that the time period of the study was more violent than any other, but rather that functional reporting tools allowed for a higher quality of more accurate data. When IPV point-of-care tools (e.g., in patient care record documentation) are developed, adopting similar principles of functionality, automation, and feedback could be beneficial. The simultaneous development of innovative and functional education, training, and infrastructure would likely support substantial progress at the intersection of paramedicine and IPV.

Many paramedics do an excellent job caring for patients within challenging conditions, but challenges persist. By utilizing the action items outlined here as an initiatory guide, paramedics and paramedic services may begin to improve care for survivors of IPV. Using established implementation or knowledge mobilization processes [58–60] to deploy and evaluate specific action items from this research could inform strong-footed next steps in the right direction. Future work should also investigate the practitioner perspective of paramedics. Study of multiple perspectives could provide a multi-faceted approach to develop well-rounded evidence-based guidance for paramedics and paramedic services.

### Limitations

This work had several limitations. Despite being the first study of to engage with survivors of IPV about their experiences with paramedics, the sample size was small. While no specific sample size was targeted and rich data was collected, the experiences and perspectives are limited to those of the participants and other perspectives are likely to exist. There were substantial challenges engaging with and interacting with survivors of IPV who had been seen by paramedics. These challenges included advertising the study through means that were not likely to increase the risk of violence to potential participants. Further, there was potential for bias among the participant group. Survivors of IPV who had negative experiences with paramedics may have been more likely to engage in this research than those who had positive experiences with paramedics. Participant safety was paramount, and decisions were made that limited reach but prioritized safety. Community partners (e.g., community services, emergency shelters) were essential in engaging with participants. However, it was also paramount to ensure survivors of IPV did not feel coerced or forced to participate in research when being promoted through community partners. Although common patterns emerged, alternative experiences are sure to exist. Future research should attempt to continue to learn from the experiences of survivors of IPV within the context of paramedicine and consider bias potential in design and recruitment processes.

## Conclusion

IPV continues as an unacceptable reality causing considerable damage to the holistic health of survivors. This research shows that survivors of IPV are being attended to by paramedics. If paramedicine is able to respond to and address the challenges faced by survivors of IPV and adjust with functional solutions, then a future where paramedics function as expert resources to support and provide services for survivors of IPV is achievable. Future work should aim to compliment this work from the patient perspective by learning from the community services, and paramedic perspectives, respectively. Further, proper implementation research should be conducted to trial education, training, and infrastructure advances as they are rolled out. The opportunity for growth within paramedicine is immense and the products of growth at this intersection could lead to improved outcomes for survivors of IPV.

## Electronic supplementary material

Below is the link to the electronic supplementary material.

Supplementary Material 1

## Data Availability

The data generated and analysed during the current study are not publicly available or available from the corresponding author on reasonable request due the inherent privacy requirements. Inquiries related to the data from this study should be directed to the corresponding author (RAM).
