# Supplementary material for: “The questions made me realize how many times I could have been saved and removed from that situation”: The experiences of patients attended to by paramedics for intimate partner violence, and actionable implementations for paramedicine
Source: BMC Womens Health. 2025 May 26;25:254. doi: 10.1186/s12905-025-03753-9 (PMC12105233; doi:10.1186/s12905-025-03753-9)
Supplement: Supplementary file 1 — Supplementary Material 1 [file 12905_2025_3753_MOESM1_ESM.pdf]

THE UNIVERSITY OF BRITISH COLUMBIA
Okanagan Campus

Qualitative Interviews with Survivors of Intimate Partner Violence Seen by Paramedics

Semi-Structured Interview Guide

This guide is intended to be used as a framework to guide semi-structured interviews for University of British Columbia Okanagan Behavioral Research Ethics Board approved study #H22-02770.

Section 1: Characterizing the Experience with Violence

1. Introduction

   a. Welcome and thank the participant for agreeing to participate in this study.
   b. Briefly explain the purpose of the study: to gain insights into survivors' experiences with paramedics in the context of intimate partner violence.
   c. Assure them of confidentiality and their right to withdraw at any point during the interview.
   d. Obtain informed consent for participation.

2. Overview of the Experience with Violence

   a. Explain definition of intimate partner violence.
   b. Respectfully inquire about their current and previous relationship status and their experience with intimate partner violence.
   c. Ask the participant to describe their experience with intimate partner violence, including type, specific incidents and the frequency of violence.
   d. Encourage them to share any factors (e.g., arguments, finances, pregnancy status, substances, etc.) that contributed to the escalation of violence and potential barriers they faced when seeking help.

3. Emotional and Physical Impact

   a. Ask about the emotional and physical impact of the violence on the survivor's well-being.
   b. If physical violence occurred, inquire about mechanisms (e.g., direct blows, strangulation, frequency, etc.) and inquire about signs and symptoms of brain injury (indirectly, see checklist below).
   c. Ask if the survivor was aware or ever informed they may have sustained a brain injury.

d. Explore coping mechanisms they used to manage the trauma resulting from the violence.

4. Childhood Experience

a. Were you exposed to intimate partner violence during in childhood?
b. Did you experience abuse or neglect during your childhood?
c. Did you experience any other adverse childhood experiences?

Section 2: Characterizing the Service Received by Paramedics

1. Engagement with Paramedics
    a. Inquire about the survivor's previous interactions with paramedics in response to incidents of intimate partner violence.
    b. Explore positive and negative experiences.
    c. Explore the circumstances surrounding these encounters (e.g., who called, why, an excessive situation of "normal violence", etc.) and whether they felt adequately supported (e.g., did paramedics attend, was it prompt, was safety addressed, etc.).

2. Perceived Response from Paramedics
    a. Ask about the survivor's perception of how emergency health services responded to their situation (e.g., Listen, Inquire about needs, Validate, Enhance safety, Supports [LIVES] principles, etc.).
    b. Prompt them to discuss the level of empathy, sensitivity, and understanding demonstrated by paramedics during the encounter.
    c. Enquire about hands-off and hands-on assessment, and what areas of concern were highlighted.

3. Identification of Intimate Partner Violence
    a. Inquire whether the paramedics recognized and appropriately addressed the signs of intimate partner violence.
    b. Was their partner present? Were steps taken to remove the partner from the situation?
    c. Ask if there were any missed opportunities for intervention or support during these encounters.

4. Outcomes from Accessing Paramedics

a. Inquire what outcomes occurred (e.g., transport by paramedics, non-transport, transport to what locations, referrals, police interference, etc.).
b. Inquire who chose the outcome and why (e.g., paramedics, police, patient, etc.).
c. Inquire about short-term outcomes (hours to week).
d. Inquire about long-term outcomes (weeks to years).
e. Inquire about if the survivor was able to leave the situation of violence or was provided with harm reduction strategies.
f. Did paramedics play a role in facilitating a better or worse outcome?

Section 3: Desired Services from Paramedics

1. Supportive Services
   a. Explore the survivor's expectations of support from emergency health services during instances of intimate partner violence.
   b. Ask about the types of resources they believe paramedics should be equipped with to assist survivors effectively.

2. Training and Education
   a. Inquire about the survivor's perspective on the adequacy of paramedics' training and knowledge regarding intimate partner violence.
   b. Prompt them to suggest any specific areas of training that might enhance the paramedics' ability to respond appropriately.

3. Coordination with Other Services
   a. Discuss the survivor's thoughts on better coordination between paramedic services and other agencies (e.g., law enforcement, social services, shelters) to address intimate partner violence effectively.
   b. Ask about their experiences with existing collaborations and suggestions for improvement.

4. Recommendations for Improvement
   a. Allow the survivor to share any additional recommendations or suggestions for how paramedic services can better support individuals experiencing intimate partner violence.

Section 4: Concluding

1. Debriefing and Support
    a. Offer the participant an opportunity to debrief and express any emotions that may have been triggered during the interview.
    b. Provide information on available support services, including counseling or support groups, if they are interested.

2. Closing
    a. Express gratitude for their participation and willingness to share their experiences.
    b. Reiterate the confidentiality of their responses and how their insights will contribute to improving emergency health services for survivors of intimate partner violence.
    c. Inform them about any follow-up steps, such as sharing the study findings if they wish to receive them.

Note: It's essential to approach this topic with sensitivity and empathy, ensuring that participants are emotionally supported throughout the interview process. Always prioritize the well-being and comfort of the survivors and be prepared to provide appropriate resources if needed.
